# Management of Postoperative Pain Following Primary Total Knee Arthroplasty: A Level I Evidence-Based Bayesian Network Meta-Analysis

**DOI:** 10.3390/ph18040556

**Published:** 2025-04-09

**Authors:** Filippo Migliorini, Marcel Betsch, Tommaso Bardazzi, Giorgia Colarossi, Hani Ayad Mohamed Elezabi, Arne Driessen, Frank Hildebrand, Mario Pasurka

**Affiliations:** 1 Department of Orthopaedic and Trauma Surgery, Academic Hospital of Bolzano (SABES-ASDAA), Via Lorenz Böhler 5, 39100 Bolzano, Italy; 2 Department of Life Sciences, Health, and Health Professions, Link Campus University, Via del Casale di San Pio V, 00165 Rome, Italy; 3 Department of Orthopaedic, Trauma and Reconstructive Surgery, RWTH University Hospital of Aachen, 52074 Aachen, Germany; 4 Department of Orthopaedic, Trauma and Reconstructive Surgery, University Hospital of Erlangen, 91054 Erlangen, Germany; 5 Department of Internal Medicine, Rhein-Maas Klinikum, 52146 Würselen, Germany; 6 Department of Anesthesia, Eifelklinik St. Brigida, 52152 Simmerath, Germany; 7 Department of Orthopaedic and Trauma Surgery, Luisenhospital, 52064 Aachen, Germany

**Keywords:** total knee arthroplasty, postoperative pain, meta-analysis, orthopaedic

## Abstract

**Background:** Postoperative pain management after total knee arthroplasty (TKA) is crucial for promoting early recovery. Advances in pain management techniques have significantly improved outcomes after TKA. Recently, multimodal analgesia has emerged as a key concept in pain management following TKA, using regional anaesthesia to reduce narcotic use and minimise narcotic-related side effects. This Bayesian network meta-analysis compared different treatment options for the management of postoperative pain following primary TKA. **Methods:** This study was conducted following the 2020 PRISMA statement. In January 2025, all randomised controlled trials (RCTs) related to postoperative pain management following TKA were accessed. Pain reported on postoperative days (PODs) 1–3 was evaluated. **Results:** Data from 7199 patients were retrieved. Of these, 63.2% (4232 of 6691) were women, and the mean age was 66.7 ± 3.1 years. The mean length of follow-up was 10.2 ± 18.3 weeks. At baseline, comparability was confirmed for age (*p* = 0.1), BMI (*p* = 0.8), and visual analogue scale (VAS, *p* = 0.1). On POD 1, single-shot SNB/three-in-one block was associated with a lower VAS, followed by continuous intra-articular analgesia/local infiltration analgesia (LIA)/posterior capsule infiltration (PCI) and continuous femoral nerve block (FNB)/intermittent SNB. On POD 2, continuous intra-articular analgesia/LIA/PCI was associated with a lower VAS, followed by continuous FNB/PCI and single-shot femoral triangle block (FTB)/single-shot infiltration between the popliteal artery and capsule of the knee (IPACK). On POD 3, continuous ACB was associated with a lower VAS, followed by continuous intra-articular analgesia/LIA/PCI and continuous FNB/PCI. **Conclusions:** Continuous intra-articular analgesia/LIA/PCI was associated with the best pain control following primary TKA. Multimodal analgesia, which incorporates peripheral nerve blockade and periarticular injections, has become a key concept in contemporary pain management following TKA.

## 1. Introduction

Total knee arthroplasty (TKA) is a widely performed surgical procedure for patients with end-stage osteoarthritis (OA) or rheumatic arthritis to improve mobility and alleviate joint pain [1,2,3,4]. TKA is one of the most effective surgeries in musculoskeletal medicine [5,6,7,8,9]. However, despite its effectiveness, TKA often results in moderate to severe postoperative pain, which can be challenging to manage [10]. In some cases, patients experience extreme immediate postoperative pain [11], which can significantly hinder rehabilitation efforts, reduce patient satisfaction, and adversely impact the overall outcomes of the procedure. Severe postoperative pain can lead to prolonged hospital stays, increased readmissions, and higher opioid consumption, often accompanied by nausea and vomiting. These factors reduce patient satisfaction and increase healthcare costs [11,12]. Therefore, effective postoperative pain management is crucial for prompting early recovery and improving patient outcomes. In the past, perioperative pain management for TKA primarily relied on opioids [13]. However, the use of opioids is associated with adverse effects, such as risk of addiction and adverse side effects, limiting their routine application in clinical practice. Advances in pain management techniques, especially in the last two decades, have significantly improved the outcomes and practice of TKA. Recently, multimodal analgesia has emerged as a key concept in pain management following TKA. This approach incorporates techniques such as local infiltration analgesia (LIA), posterior capsule infiltration (PCI), peripheral nerve blocks such as adductor canal block (ACB), femoral nerve block (FNB) and sciatic nerve block (SNB), intravenous patient-controlled analgesia (PCA), epidural anaesthesia, and the use of various pain medications [10,11,14].

There is a growing trend toward multimodal approaches using regional anaesthesia to reduce narcotic use and minimise narcotic-related side effects [11]. However, these techniques also have limitations, such as suboptimal pain control and unwanted side effects, and no gold standard has been established yet. Therefore, this Bayesian network meta-analysis aimed to compare the different treatment options in managing postoperative pain following primary TKA.

## 2. Methods

### 2.1. Search Strategy

This Bayesian network meta-analysis was conducted according to the PRISMA extension statement for reporting systematic reviews incorporating network meta-analyses of healthcare interventions [15]. The following framework (PICOTD) was used for the search:

- P (Problem): TKA;
- I (Intervention): postoperative pain control;
- C (Comparison): different strategies to manage pain control;
- O (Outcomes): visual analogue scale;
- T (Timing): hospitalisation;
- D (Design): randomised controlled trials.

PubMed, Web of Science, and Embase were accessed in January 2025 without additional filters or temporal constraints. The Medical Subject Headings (MeSHs) used in the database are reported as Supplementary Materials.

### 2.2. Eligibility Criteria

All randomised controlled trials (RCTs) concerning postoperative pain management following TKA were considered. Eligible studies were required to be published in peer-reviewed journals. According to the authors’ capabilities, only articles in the following languages were considered: Italian, Spanish, German, English, or French. Only studies with levels I to II of evidence, according to the Oxford Centre of Evidence-Based Medicine (OCEBM) [16], were considered. Studies that evaluated other non-pharmacological analgesia modalities were not considered. Opinions, letters, editorials, and reviews were excluded. Additionally, studies involving animals, computational analyses, biomechanical assessments, in vitro experiments, or cadaveric research were disregarded. Only RCTs concerning pain management in TKA were included. Studies evaluating monocompartmental arthroplasty or those in revision settings were not eligible. Only studies reporting data on the visual analogue scale (VAS) [17] for each postoperative day (POD) were included.

### 2.3. Outcomes of Interest

Three authors (H.A.M.E, G.C., and T.B.) conducted data extraction. For each study, the following generalities were collected: author, year of publication, journal, study design, and length of follow-up. The following data at baseline were retrieved: number of patients, number of women, mean age, and mean BMI. Data concerning VAS were collected for PODs 0, 1, 2, and 3 and at discharge. Extraction was performed using Microsoft Office Excel version 16.0 (Microsoft Corporation, Redmond, WA, USA).

### 2.4. Methodology Quality Assessment

Three authors (H.A.M.E., G.C., and T.B.) performed the methodological quality assessment using the revised Risk of Bias assessment tool (RoB2) [18,19] of the Cochrane tool for assessing the Risk of Bias in randomised trials [20]. The following endpoints were considered: bias arising from the randomisation process, bias due to deviations from intended interventions, bias due to missing outcome data, bias in measuring the outcome, and bias in selecting the reported result.

### 2.5. Statistical Analysis

The statistical analysis was performed by the main author (F.M.). STATA Software/MP (version 15, StataCorporation, College Station, TX, USA) was used for the statistical analyses. The baseline comparability was assessed using analysis of variance (ANOVA), with *p*-values > 0.1 considered satisfactory. The network meta-analyses were performed through the STATA routine using the inverse variance method for Bayesian hierarchical random-effects model analysis. The standardised mean difference (STD) was calculated for continuous data. The overall inconsistency was evaluated through the equation for global linearity via the Wald test. If the *p*-value > 0.1, the null hypothesis cannot be rejected, and the consistency assumption is accepted at the overall level of each treatment. Edge plots were performed to display direct and indirect comparisons and respective statistical weights. Interval plots were performed to rank each treatment according to their estimated effect. Both confidence (CI) and percentile (PrI) intervals were set at 95% in each interval plot. Funnel plots were performed to investigate the risk of bias related to each comparison. Greater plot asymmetry indicates greater data variability and is associated with a greater risk of bias.

## 3. Results

### 3.1. Search Result

**Figure 1:**
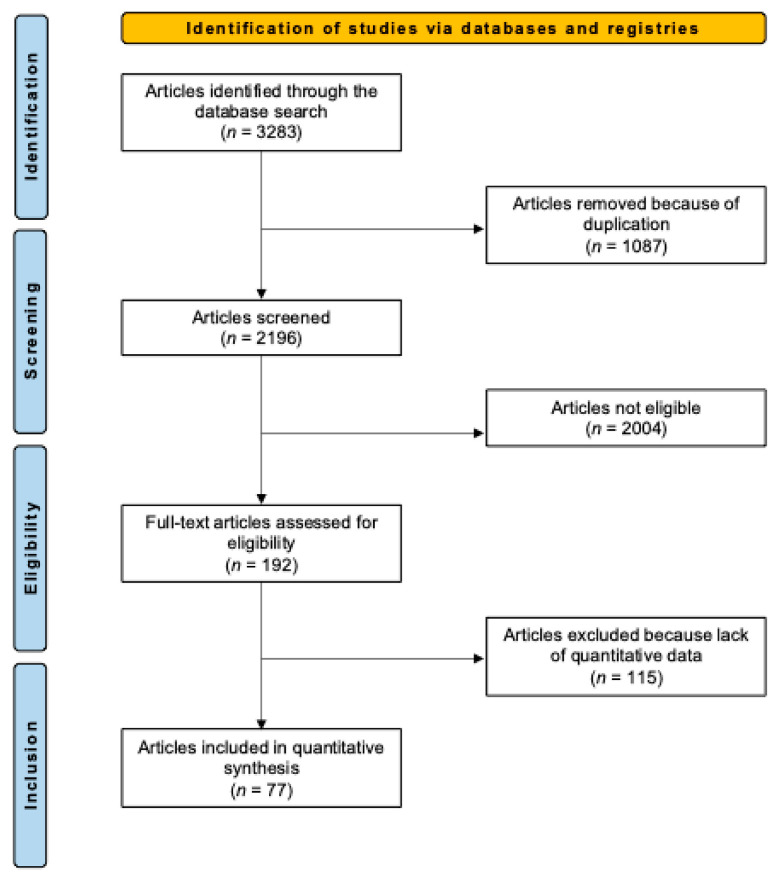
Flowchart of the literature search.

The systematic literature search resulted in the identification of 3283 articles. After removing duplicates, the abstracts of 2196 articles were screened for eligibility. A total of 2004 articles were excluded for the following reasons: mismatch with the predefined study design criteria (*n* = 1123), full-text unavailability (*n* = 725), and language limitations (*n* = 156). Of the remaining 192 studies, another 115 were excluded after full-text evaluation. Consequently, a final selection of 77 studies [21,22,23,24,25,26,27,28,29,30,31,32,33,34,35,36,37,38,39,40,41,42,43,44,45,46,47,48,49,50,51,52,53,54,55,56,57,58,59,60,61,62,63,64,65,66,67,68,69,70,71,72,73,74,75,76,77,78,79,80,81,82,83,84,85,86,87,88,89,90,91,92,93,94,95,96] was included in this systematic review. The results of the literature search are shown in Figure 1.

### 3.2. Methodological Quality Assessment

**Figure 2:**
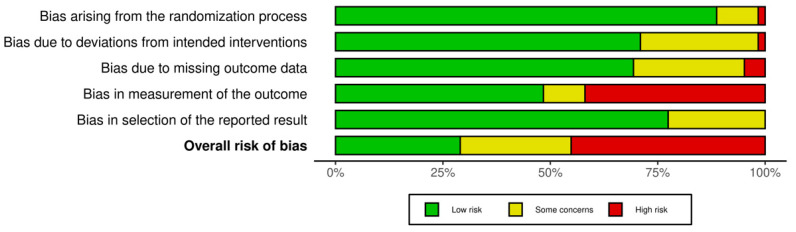
Methodological quality assessment.

The Cochrane Risk of Bias Assessment tool (ROB 2) was used to evaluate the 77 included RCTs. The analysis suggested a generally low to moderate risk of bias in the first three and the last domains: randomisation process, deviation from intended intervention, missing data, and selection of the reported result. Given the lack of blinded assessors, the outcome measurement, on the other hand, was at high risk in nearly half of the RCTs. The overall RoB was estimated to be low or moderate in more than half of the included RCTs, suggesting an acceptable methodological quality. However, caution must be paid to the potential bias in the outcome measurements. Figure 2 shows the bias risk distribution across the included RCTs.

### 3.3. Patient Demographics

Data from 7199 patients were retrieved, 63.2% of whom (4232 of 6691) were women. The mean length of follow-up was 10.2 ± 18.3 weeks. The mean age was 66.7 ± 3.1 years, and the mean BMI was 28.6 ± 3.0 kg/m^2^. The ANOVA test found comparability in age (*p* = 0.1), BMI (*p* = 0.8), and VAS (*p* = 0.1) at baseline. Table 1 shows the generalities and demographics of the studies.

### 3.4. Outcomes of Interest

Sixty-two RCTs (5856 patients) reported data on POD 1 [21,22,23,25,26,27,28,29,30,31,34,35,36,38,39,42,45,46,47,48,49,50,51,52,53,54,55,56,57,58,59,60,61,62,66,67,68,69,70,71,72,73,74,75,76,77,78,80,83,84,86,87,88,89,90,91,92,93,94,95,96]. Single-shot SNB/three-in-one block was associated with a lower VAS (SMD −0.50; 95% CI −1.98 to 0.98), followed by continuous intra-articular analgesia/LIA/PCI (SMD −0.17; 95% CI 2.13 to 1.78) and continuous FNB/intermittent SNB (SMD −0.0; 95% CI −2.13 to 2.13).

Fifty-three RCTs (5695 patients) reported data on POD 2 [23,24,25,27,31,36,37,39,40,41,42,43,44,45,47,50,51,52,53,54,55,56,58,59,60,62,63,64,65,66,69,70,71,72,73,74,77,78,79,80,81,84,86,87,88,89,90,91,92,93,94,95,96]. Continuous intra-articular analgesia/LIA/PCI was associated with a lower VAS (SMD −0.76; 95% CI 2.40 to 0.87), followed by continuous FNB/PCI (SMD −0.50; 95% CI −1.73 to 0.74) and single-shot FTB/single-shot IPACK (SMD −0.35; 95% CI −2.08 to 1.38).

Twenty-nine RCTs (2101 patients) reported data on POD 3 [21,23,27,31,32,33,36,37,42,44,45,50,51,54,55,56,58,70,76,80,81,82,85,87,88,89,90,91,95]. Continuous ACB was associated with a lower VAS (SMD −1.00; 95% CI −3.62 to 1.62), followed by continuous intra-articular analgesia/LIA/PCI (SMD −0.70; 95% CI −2.51 to 1.11) and continuous FNB/PCI (SMD −0.52; 95% CI −2.33 to 1.29). Edge, funnel, and interval plots of each POD are reported as Supplementary Materials.

## 4. Discussion

Multimodal analgesia is a key concept in pain management following TKA. However, there is still debate regarding the most effective combination of techniques. According to the findings of the present Bayesian network meta-analysis, continuous intra-articular analgesia/LIA/PCI was associated with superior pain control after primary TKA. The lowest VAS scores were observed with single-shot SNB/three-in-one block on postoperative day 1, for continuous intra-articular analgesia/LIA/PCI on postoperative day 2, and continuous ACB on postoperative day 3.

Traditional general anaesthesia combined with postoperative epidural and patient-controlled opioid analgesia is associated with a high rate of undesirable adverse effects. In contrast, the emerging concept of multimodal anaesthesia for TKA offers superior pain control, minimises opioid-related side effects, enhances patient satisfaction, and reduces the risk of postoperative complications [97]. In addition to oral opioid and non-opioid medications during the perioperative and postoperative period, multimodal anaesthesia integrates elements of pre-emptive analgesia, neuraxial perioperative anaesthesia, peripheral nerve blockade (PNB), and periarticular injections (PAI) [97].

However, despite the availability of multimodal treatment options for pain management following TKA, there is still no consensus on the optimal method [13,14]. PNB, LIA, and PCA are the most commonly used techniques. PCA involves using a programmable device tailored to the analgesic, the patient’s physical characteristics, and baseline pain levels. A small amount of analgesic can be delivered when patients press a button to administer it as needed. While opioids, such as morphine, fentanyl, and hydromorphone, are commonly used in PCA, it is associated with some adverse effects [98]. However, these effects are generally less severe than those caused by conventional opioid treatment [99]. Additionally, PCA encourages early mobilisation and reduces the length of hospital stays after TKA [13]. Based on a systematic review and meta-analysis, the International Consensus on Anesthesia-Related Outcomes after Surgery (ICAROS) group recommends PNB use in THA/TKA for improved outcomes [100]. Among PNB, femoral nerve block (FNB) has been widely accepted as the gold standard for pain relief after TKA. It provides adequate postoperative analgesia and contributes to long-term functional recovery in patients undergoing TKA [101]. However, FNB also reduces quadriceps muscle strength, which limits knee extension [102,103] and is associated with potentially serious complications, such as blood vessel and nerve damage [104]. Because the knee is innervated by several nerves, including the femoral, sciatic, obturator, saphenous, and lateral femoral cutaneous nerve, pain in the posterior aspect of the knee is not adequately reduced by FNB alone. Combining FNB with a sciatic nerve block (SNB) could address this limitation effectively. However, a combination of SNB with FNB may cause postoperative muscle weakness and may delay rehabilitation in the early postoperative period. Besides FNB and SNB, ACB is becoming increasingly popular as it provides postoperative pain relief as effectively as FNB but without impairing quadriceps muscle strength [103,105]. With ACB, it may be possible to block the saphenous nerve while sparing the major motor branches of the femoral nerve [13]. Compared with FNB, patients receiving ACB experience better quadriceps muscle strength, improved early rehabilitation, longer ambulation distances, and shorter hospital stays [79,106]. However, ACB does not adequately relieve lateral knee pain in the early stages [79] and remains a relatively new regional anaesthesia technique for TKA, requiring further clinical and scientific evaluation. In a systematic review and meta-analysis by Sercia et al., continuous ACB significantly reduced 48 h pain scores but did not significantly decrease opioid consumption [107]. LIA has gained significant interest in recent years given its simplicity, low associated risk, and reduced likelihood of systemic toxicity from local anaesthetics [13]. Additionally, LIA can address posterior knee pain by enabling injections into the posterior joint capsule (PCI: posterior capsule infiltration).

Furthermore, ultrasound-guided infiltration of local anaesthetics in the interspace between the popliteal artery and the posterior capsule of the knee (IPACK) is a novel regional anaesthetic technique for posterior knee analgesia that has shown promising results [108]. LIA is regarded as a promising method for pain management due to its ability to facilitate early mobilisation without compromising quadriceps muscle strength [109]. For this reason, combining an LIA with FNB may be a more acceptable approach than combining SNB with FNB for pain management following TKA [14]. A meta-analysis by Zhang et al. demonstrated that LIA was as effective as FNB regarding VAS scores for pain control at 24, 48, and 72 h, total morphine consumption, range of motion, knee society scores, complications, and length of hospital stay [110]. Furthermore, LIA significantly improved postoperative pain relief and reduced opioid consumption compared with ACB [111]. In contrast to the present findings, a meta-analysis by Wang et al. showed that single-shot FNB might provide better pain relief in the early postoperative period compared with single-shot periarticular multimodal drug injection (PMDI)/LIA. At the same time, continuous PMDI/LIA offered postoperative analgesia comparable to continuous FNB [112]. When comparing the efficacy of LIA and SNB combined with single-shot and continuous FNB, Tanikawa et al. found that SNB was more effective than LIA in reducing pain immediately after surgery [81]. However, consistent with the present findings, SNB was less effective than LIA at 24 h post-surgery [81]. Interestingly, a recent network meta-analysis suggested that ACB combined with IPACK may be the optimal analgesic regimen for TKA patients [113]. The analysis included and compared FNB, ACB, IPACK, and genicular nerve block (GNB). ACB + IPACK was the most effective regimen for resting pain and movement pain relief (78% and 87%, respectively) and for reducing opioid consumption (90%) at 48 h. Meanwhile, FNB combined with IPACK was the most efficacious option for resting pain relief (42%) and reducing opioid consumption (68%) at 24 h. GNB was the most effective option for movement pain relief at 24 h (94%) [113]. These conflicting results may be attributed to the different analgesic methods included in the analysis.

This study has several limitations. The present Bayesian network meta-analysis encompasses a wide range of interventions. The strength of Bayesian network meta-analysis is its capability to integrate a variety of treatments while maintaining consistency and validity through the concept of transitivity. In the present investigation, the authors carefully examined the comparability of studies, ensuring that the included treatments were sufficiently similar regarding their overall treatment objectives, population characteristics, and methodological rigour. Using a random-effects model accounts for variability between studies, including differences in treatment regimens, dosages, and patient populations. This model explicitly acknowledges and incorporates between-study heterogeneity, thereby enabling the synthesis of diverse treatment arms without compromising the validity of the pooled estimates. While the authors recognise that combining diverse treatments requires careful consideration, the Bayesian framework offers a robust methodology for pooling results, provided that the studies meet established methodological criteria. This meta-analytic approach was intentionally designed to maximise the inclusivity of relevant studies, ensuring a comprehensive dataset for the network meta-analyses. A key principle of Bayesian network meta-analysis is that its reliability and robustness directly depend on the quantity and connectivity of the available data. By minimising restrictive eligibility criteria, the present study aimed to include the broadest possible range of high-quality RCTs, thereby enhancing the statistical power and transitivity of the network. This approach is particularly critical in Bayesian frameworks, where prior distributions and borrowing of strength across comparisons require a well-connected and sufficiently populated network to yield stable and generalisable results. Only short-term pain relief was evaluated in this analysis, making it unclear which modality provides the best long-term clinical outcome. Additionally, this meta-analysis included multiple studies that are heterogenous and differ in several aspects, such as participant characteristics (age, sex, activity, BMI, etc.), single- or multicentre study designs, cohort size (small-, medium- and large-sized studies), and the number and types of analyses conducted. Another limitation is that ACB can be categorised into proximal and distal blocks, which produce different effects [114]. However, these distinctions were not considered in the present analysis. Moreover, the concentration and volume of local anaesthetic varied between studies, potentially affecting the analgesic outcomes. This study compared different treatment options for managing postoperative pain following primary TKA. Other aspects, such as the use of various drugs with differing times of onset and duration of effect, mechanisms of action, and routes of administration, were not included in this study. Additionally, the type of implants (e.g., short- or standard-stem and dual- or single-mobility) and related variations (e.g., cementation and surgical access) were not considered. Furthermore, there were differences in postoperative rehabilitation protocols and concomitant procedures performed across studies. These differences were not accounted for in the final analysis, although they could influence the results.

The strength of the present study is the analysis of various analgesic methods. We included several local infiltration methods, peripheral nerve blocks, intravenous patient-controlled analgesia, epidural anaesthesia, and the use of pain medications.

## 5. Conclusions

Continuous intra-articular analgesia, LIA, or PCI was associated with the best pain control following primary TKA. However, there appears to be a shift toward multimodal approaches, using regional anaesthesia to minimise narcotic consumption. Multimodal analgesia, incorporating peripheral nerve blockade and periarticular injection elements, has become a cornerstone in pain management following TKA.

## Figures and Tables

**Table 1:** Characteristics and patient baseline of the included studies (LIA: local infiltration analgesia; PCI: posterior capsule infiltration; ACB: adductor canal block; FNB: femoral nerve block; SNB: sciatic nerve block; PCA: patient-controlled analgesia; FTB: femoral triangle block; IPACK: infiltration between the popliteal artery and capsule of the knee).

Author and Year	Journal	Treatment Group	Patients (*n*)	Women (*n*)	Mean Age	Mean BMI
Adams et al., 2002 [21]	*Eur J Anaesthesiol*	Three-In-One Block	21	16	70.0	
		EDA	21	14	69.0	
		PCA	21	14	69.0	
Akesen et al., 2021 [22]	*Acta Orthop Traumatol Turc*	Single-Shot IPACK	20	17	67.5	31.8
		Single-Shot GNB	20	16	68.0	33.5
		PCA	20	17	63.0	34.4
Albrecht et al., 2013 [23]	*Clin Orthop Relat Res*	Continuous FNB	28	13	61.0	32.0
		Continuous FNB	32	14	63.0	32.0
		Sham Block and Single-Shot FNB	33	17	63.0	31.0
Al-Zahrani et al., 2015 [24]	*J Arthroplasty*	Continuous EDA	25	18	60.0	33.0
		Continuous FNB and Single-Shot SNB	25	17	62.0	33.0
Ardon et al., 2016 [25]	*J Clin Anesth*	Continuous FNB and Intermittent SNB	45	31	67.7	
		Continuous ACB and Intermittent SNB	45	31	64.9	
Ashraf et al., 2013 [26]	*Knee*	Single-Shot FNB	22			
		LIA	20			
Bagry et al., 2008 [27]	*Reg Anesth Pain Med*	Continuous Lumbar Plexus Block and Continuous SNB	6	3	69.0	
		PCA	6	4	74.0	
Bali et al., 2016 [28]	*J Clin Anesth*	Single-Shot Fascia Iliaca Block	33	18	63.3	
		PAI	35	21	61.7	
Baranovic et al., 2011 [29]	*Coll Antropol*	Continuous FNB	35	16	69.0	26.0
		PCA	36	18	70.0	27.2
Campbell et al., 2008 [30]	*Eur J Anaesthesiol*	Continuous EDA	31	17	70.0	30.8
		Continuous Lumbar Plexus Block	29	15	72.0	29.1
Canbek et al., 2019 [31]	*Acta Orthop Traumatol Turc*	Continuous ACB	63	48	66.9	31.4
		Single-Shot ACB	60	50	67.1	32.3
Cappelleri et al., 2011 [32]	*Reg Anesth Pain Med*	Continuous Lumbar Plexus Block and Continuous SNB	19	13	69.0	29.0
		Continuous Lumbar Plexus Block and Sham SNB and Single-Shot SNB	19	11	67.0	28.0
Cartli et al., 2010 [33]	*Br J Anaesth*	Continuous FNB and Sham Peri- and Intra-articular Analgesia	20	14	71.1	27.0
		Peri- and Intra-articular Analgesia and Sham Block	20	15	70.8	28.5
Carvalho et al., 2012 [34]	*Open Journal of Anesthesiology*	Continuous FNB and Single-Shot SNB	25	16	65.0	29.9
		Continuous FNB	25	20	68.0	28.1
Casati et al., 2005 [35]	*Anesth Analg*	Continuous FNB and Single-Shot SNB	20	12	66.0	
		Continuous FNB and Single-Shot SNB	20	13	65.0	
		Continuous FNB and Single-Shot SNB	20	11	70.0	
Chan et al., 2012 [36]	*Acta Anaesthesiol Taiwan*	Single-Shot FNB	20	16	68.1	
		Single-Shot FNB	21	15	67.3	
		Sham Block	20	15	70.9	
		Sham Block	21	14	71.8	
Chan et al., 2013 [37]	*J Arthroplasty*	Continuous FNB	65	53	66.4	27.7
		Single-Shot FNB	69	57	66.1	26.7
		PCA	66	53	64.7	26.3
Chaumeron et al., 2013 [38]	*Clin Orthop Relat Res*	Continuous FNB and Sham PAI	30	23	66.6	
		PAI and Sham FNB	30	16	67.3	
Cicekci et al., 2019 [39]	*Sao Paulo Med J*	PAI	40	30	68.5	32.0
		Single-Shot ACB	39	28	69.1	32.5
Elkassabany et al., 2016 [40]	*Anesth Analg*	Continuous FNB	31	19	65.0	32.0
		Continuous ACB	31	22	63.0	31.0
Elkassabany et al., 2019 [41]	*Bone Joint J*	Continuous ACB and PAI	51	22	66.5	31.2
		Continuous ACB and PAI	52	18	62.2	31.9
		PAI and Single-Shot ACB	53	16	63.9	31.5
Fritze et al., 2009 [42]	*Schmerz*	Continuous EDA	18	15		
		Continuous FNB	17	16		
		Continuous SNB	17	11		
Gi et al., 2014 [43]	*J Anesth*	PAI and Single-Shot FNB	25	24	77.0	27.0
		Sham PAI and Single-Shot FNB and Single-Shot SNB	24	21	78.0	28.0
Good et al., 2007 [44]	*Am J Orthop (Belle Mead NJ)*	Single-Shot FNB	22	8	70.0	
		Sham Block	20	8	70.0	
Grosso et al., 2018 [45]	*J Bone Joint Surg Am*	Single-Shot ACB	53	39	69.0	30.2
		PAI	51	33	73.0	29.8
		PAI and Single-Shot ACB	51	37	71.0	30.4
Kanadli et al., 2018 [46]	*Minerva Anestesiol*	Single-Shot Fascia Iliaca Block	45	31	62.6	30.0
		Single-Shot FNB	45	37	66.9	28.7
Kovalak et al., 2015 [47]	*Acta Orthop Traumatol Turc*	Continuous FNB and PCI	32	30	69.5	36.7
		PAI and PCI	28	24	66.9	34.0
Kulkarni et al., 2019 [48]	*J Arthroplasty*	Single-Shot ACB	50	33	67.4	
		PAI	50	33	67.7	
Kurosaka et al., 2015 [49]	*J Arthroplasty*	LIA	22	19	75.6	26.3
		Continuous FNB	23	19	77.5	26.7
Kutzner et al., 2015 [50]	*Orthopade*	Continuous Intra-articular Analgesia	60	39	70.5	
		Continuous FNB	60			
Li et al., 2017 [51]	*Zhongguo Xiu Fu Chong Jian Wai Ke Za Zhi*	Continuous ACB	30			
		Single-Shot ACB	30			
Li et al., 2020 [52]	*Int Orthop*	Sham PAI and Single-Shot ACB and Single-Shot AFCNB	80	60	66.6	25.7
		PAI and Sham Block	80	60	65.2	25.3
Li et al., 2020 [53]	*J Arthroplasty*	LIA and Single-Shot ACB and Single-Shot AFCNB and Single-Shot IPACK	50	33	66.3	24.8
		LIA and Single-Shot ACB and Single-Shot IPACK	50	40	66.8	24.7
		LIA and Single-Shot ACB and Single-Shot AFCNB	50	32	66.4	24.8
		LIA and Single-Shot ACB	50	31	65.6	25.0
Li et al., 2022 [54]	*J Knee Surg*	Single-Shot FTB and Single-Shot IPACK	40	25	67.8	25.4
		Intra-articular Analgesia and Sham Block	40	22	70.8	24.3
Long et al., 2006 [55]	*J Knee Surg*	Continuous EDA and Intra-articular Analgesia	35	20	69.0	
		Continuous FNB and Intra-articular Analgesia	35			
Luo et al., 2022 [56]	*BMC Musculoskelet Disord*	LIA and Sham ACB	30	22	65.3	24.9
		LIA and Single-Shot ACB	30	23	65.4	24.8
Macrinici et al., 2017 [57]	*Reg Anesth Pain Med*	Sham FNB and Single-Shot ACB	49	30	67.0	31.5
		Sham ACB and Single-Shot FNB	49	31	67.0	31.7
Marino et al., 2019 [58]	*J Arthroplasty*	Continuous FNB and PAI	33	15	62.3	32.6
		PAI	32	17	64.2	33.1
Memtsoudis et al., 2014 [59]	*Int Orthop*	Patient-controlled EDA and Single-Shot FNB	30	17	64.4	28.4
		Patient-controlled EDA and Single-Shot ACB	29	16	64.4	28.4
Mistraletti et al., 2006 [60]	*Reg Anesth Pain Med*	Continuous FNB and Continuous SNB	8	2	67.3	29.3
		Continuous EDA	8	2	64.0	28.8
		PCA	8	4	70.5	27.5
Moreno et al., 2022 [61]	*Anaesthesiol Intensive Ther*	Continuous FNB	25	15	63.0	30.1
		LIA	25	14	65.0	29.3
Mu et al., 2022 [62]	*J Healthc Eng*	Single-Shot ACB	35	28	66.6	25.4
		Single-Shot ACB	35	25	66.4	25.0
Mudumbai et al., 2013 [63]	*Clin Orthop Relat Res*	Continuous FNB and PAI	102	4	66.0	33.0
		Continuous ACB and PAI	66	5	65.0	33.0
Ng et al., 2012 [64]	*J Arthroplasty*	Continuous FNB and Sham PAI	16	14	70.0	
		PAI and Sham Block	16	14	70.0	
Nicolino et al., 2020 [65]	*J Arthroplasty*	Intra-articular Analgesia and Single-Shot SNB	34	23	72.0	31.0
		Intra-articular Analgesia and Sham Block	36	27	72.6	30.1
Paauwe et al., 2008 [66]	*Anaesthesia*	Continuous FNB and Intermittent FNB	12	8	71.5	29.1
		Continuous FNB and Intermittent FNB	12	7	68.3	29.9
		Continuous FNB and Intermittent FNB	12	8	68.5	28.3
Parvataneni et al., 2007 [67]	*J Arthroplasty*	PAI	31	14	68.5	29.0
		Single-Shot FNB	29	15	70.5	29.4
Patterson et al., 2020 [68]	*J Arthroplasty*	Continuous ACB and Single-Shot IPACK	35	21	67.0	31.0
		Continuous ACB and Sham IPACK	34	21	68.0	30.0
Rousseau-Saine et al., 2018 [69]	*Anesth Analg*	Continuous ACB	30	21	69.0	34.0
		Sham Block	30	20	67.0	33.0
Salinas et al., 2006 [70]	*Anesth Analg*	Single-Shot FNB	18	11	67.0	32.0
		Continuous FNB	18	7	68.0	31.0
Sankineani et al., 2017 [71]	*Musculoskelet Surg*	Single-Shot ACB	100		67.0	
		PAI and Single-Shot ACB	100		65.0	
Sankineani et al., 2018 [72]	*Eur J Orthop Surg Traumatol*	Single-Shot ACB and Single-Shot IPACK	60	22	60.0	
		Single-Shot ACB	60	18	61.0	
Shah et al., 2014 [73]	*J Arthroplasty*	Intermittent ACB and Intra-articular Analgesia	48	35	68.3	29.5
		Intermittent FNB and Intra-articular Analgesia	50	36	65.9	30.5
Shah et al., 2015 [74]	*J Arthroplasty*	Continuous ACB	46	33	68.3	29.6
		Single-Shot ACB	39	32	66.3	30.3
Sites et al., 2004 [75]	*Anesth Analg*	ITM	20	11	65.0	
		Single-Shot FNB	20	10	63.0	
Stathellis et al., 2015 [76]	*Knee Surg Sports Traumatol Arthrosc*	Continuous FNB and Single-Shot SNB	25	15	67.4	
		Continuous Intra-articular Analgesia and PAI	25	16	69.4	
Tak et al., 2020 [77]	*Musculoskelet Surg*	Single-Shot ACB	58	37	64.1	26.6
		Continuous ACB	57	38	63.3	26.0
		Single-Shot ACB and Single-Shot IPACK	56	29	65.5	26.0
Talmo et al., 2018 [78]	*J Arthroplasty*	PCI and Single-Shot FNB	161	73	62.3	30.1
		PAI and Sham Block	151	81	62.0	30.7
Tan et al., 2018 [79]	*Medicine (Baltimore)*	LIA and Single-Shot ACB	100	56	64.2	26.1
		LIA and Single-Shot FNB	100	58	63.5	25.7
Tang et al., 2016 [80]	*J Orthop Surg (Hong Kong)*	Single-Shot FNB	15	12	65.0	
		Single-Shot FNB	15	12	66.0	
		Single-Shot FNB	15	11	64.0	
		Control	15	11	64.0	
Tanikawa et al., 2014 [81]	*J Arthroplasty*	Continuous FNB and Single-Shot SNB	23	20	72.0	24.5
		Continuous FNB and LIA	23	19	71.0	23.5
Tanikawa et al., 2017 [82]	*J Orthop Surg Res*	Continuous FNB and Single-Shot SNB	38	29	76.0	24.6
		Continuous FNB and LIA	41	30	74.0	25.0
Theodosiadis et al., 2013 [83]	*J Orthop Surg (Hong Kong)*	Single-Shot SNB and Three-In-One Block	20	12	73.0	
		Single-Shot SNB and Three-In-One Block	20	14	70.0	
Thobhani et al., 2017 [84]	*Ochsner J.*	Continuous FNB	61	38	67.0	33.0
		Continuous FNB and Single-Shot IPACK	23	14	69.0	32.0
		Continuous ACB and Single-Shot IPACK	22	14	63.0	36.0
Tsukada et al., 2014 [85]	*J Bone Joint Surg Am*	PAI	50			
		Continuous EDA	61			
Wall et al., 2017 [86]	*Bone Joint J*	Single-Shot FNB	131	80	68.2	
		PAI	131	77	68.7	
Wang et al., 2002 [87]	*Reg Anesth Pain Med*	Single-Shot FNB	15	9	67.0	
		Sham Block	15	10	66.0	
Wang et al., 2019 [88]	*J Arthroplasty*	PCI and Single-Shot ACB	45	30	64.8	25.2
		PAI	45	33	64.0	25.1
Wang et al., 2020 [89]	*Clin J Pain*	Continuous ACB	30	14	61.7	
		Continuous FTB	30	15	61.8	
Wang et al., 2022 [90]	*BMC Anesthesiol*	Patient-controlled ACB and Sham IPACK	35	29	64.2	27.8
		Patient-controlled ACB and Single-Shot IPACK	35	28	66.5	27.1
Wu et al., 2014 [91]	*Hong Kong Med J*	Continuous FNB	30	22	68.8	27.8
		PCA	30	22	68.9	28.3
Yao et al., 2019 [92]	*Medicine (Baltimore)*	Single-Shot FNB	266	195	66.9	25.7
		PCA	470	328	66.6	25.5
Zaric et al., 2006 [93]	*Anesth Analg*	Continuous EDA	23	11	67.0	
		Continuous FNB and Continuous SNB	26	15	66.0	
Zhao et al., 2019 [94]	*Med Princ Pract*	Continuous FNB	30	12	70.4	
		Continuous FNB	30	16	73.2	
		Continuous FNB	30	20	71.5	
Zinkus et al., 2017 [95]	*Med Sci Monit*	Continuous FNB and PCI	27	16	70.4	29.4
		Continuous Intra-articular Analgesia and LIA and PCI	27	25	66.9	30.3
Zoratto et al., 2021 [96]	*Can J Anaesth*	PAI and Single-Shot ACB	39	18	66.7	32.4
		PAI and Single-Shot ACB	41	19	67.5	32.8
		PAI and Sham ACB	41	19	67.5	33.2

## Data Availability

The data are contained within this article or Supplementary Materials.

## Supplementary Materials

The following supporting information can be downloaded at https://www.mdpi.com/article/10.3390/ph18040556/s1, File S1: Research Questions and Searching Strategy.
