# Supplementary material for: Management of Postoperative Pain Following Primary Total Knee Arthroplasty: A Level I Evidence-Based Bayesian Network Meta-Analysis
Source: Pharmaceuticals (Basel). 2025 Apr 9;18(4):556. doi: 10.3390/ph18040556 (PMC12030410; doi:10.3390/ph18040556)
Supplement: Supplementary file 1 [file pharmaceuticals-18-00556-s001.zip › pharmaceuticals-3513756-supplementary.pdf]

Research Question:
Postoperative pain management in TKA

Concept 1 – Problem: Postoperative pain in TKA

Keywords:
Arthroplasty
arthroplasty, replacement, knee
Knee
Knee arthroplasty
osteoarthritis
Pain
Pain, postoperative
replacement–total knee
TKA
Total knee arthroplasty

Mesh:
"Arthralgia / diagnosis"[Mesh]
"Arthralgia / etiology"[Mesh]
"Arthroplasty, Replacement, Hip"[Mesh]
"Arthroplasty, Replacement, Knee"[Mesh]
"Intraoperative Care"[Mesh]
"Intraoperative Period"[Mesh]
"Joint Capsule / innervation"[Mesh]
"Knee Joint"[Mesh]
"Knee Joint / physiopathology"[Mesh]
"Knee Joint / surgery"[Mesh]
"Osteoarthritis, Knee / pathology"[Mesh]
"Osteoarthritis, Knee / surgery"[Mesh]
"Pain, Postoperative / surgery"[Mesh]

Concept 2 - Intervention: Postoperative Analgesia

Keywords:
analgesia
pain control
pain management
Postoperative analgesia

Mesh:
"Analgesia"[Mesh]
"Analgesics"[Mesh]
"Analgesics / administration & dosage"[Mesh]
"Analgesics / therapeutic use"[Mesh]
"Anesthesia, Conduction"[Mesh]
"Chronic Pain / drug therapy"[Mesh]
"Pain / prevention & control"[Mesh]
"Pain Management"[Mesh]
"Pain Management / methods"[Mesh]
"Pain, Postoperative / drug therapy"[Mesh]
"Perioperative Care / methods"[Mesh]

Concept 3 – Comparison: Analgesia Modalities

Keywords:
analgesia / methods
Continuous
Femoral block
femoral nerve block
Intra-articular
iPACK
Lateral femoral cutaneous nerve block
LIA
Local infiltration
Local infiltration analgesia
multimodal analgesia
Multimodal infiltration
Nerve block
Periarticular drug injection
periarticular infiltration
periarticular injection
Perineural catheters
peripheral nerve block
Single injection femoral nerve block
Single shot

Mesh:
"Analgesia / methods"[Mesh]
"Analgesics, Non-Narcotic / therapeutic use"[Mesh]
"Analgesics, Opioid"[Mesh]
"Anesthesia, Epidural"[Mesh]
"Anesthesia, Local"[Mesh]
"Anesthesia, Spinal"[Mesh]
"Anesthetics, Intravenous / therapeutic use"[Mesh]
"Anesthetics, Local"[Mesh]
"Autonomic Nerve Block / methods"[Mesh]
"Catheterization, Peripheral / methods"[Mesh]
"Drug Combinations"[Mesh]
"Drug Therapy, Combination"[Mesh]
"Femoral Nerve"[Mesh]
"Infusion Pumps"[Mesh]
"Infusions, Intravenous"[Mesh]
"Injections"[Mesh]
"Injections, Intra-Articular"[Mesh]
"Lumbosacral Plexus"[Mesh]
"Nerve Block"[Mesh]
"Nerve Block / methods"[Mesh]
"Sciatic Nerve"[Mesh]

Concept 4 - Outcome: VAS

Keywords:
outcomes
VAS
visual analogue score

Mesh:
"Pain Measurement"[Mesh]
"Pain Measurement / methods"[Mesh]
"Treatment Outcome"[Mesh]
"Visual Analog Scale"[Mesh]

Searching Strategy

"Arthralgia / diagnosis"[Mesh] OR "Arthralgia / etiology"[Mesh] OR "Arthroplasty, Replacement, Hip"[Mesh] OR "Arthroplasty, Replacement, Knee"[Mesh] OR "Intraoperative Care"[Mesh] OR "Intraoperative Period"[Mesh] OR "Joint Capsule / innervation"[Mesh] OR "Knee Joint"[Mesh] OR "Knee Joint / physiopathology"[Mesh] OR "Knee Joint / surgery"[Mesh] OR "Osteoarthritis, Knee / pathology"[Mesh] OR "Osteoarthritis, Knee / surgery"[Mesh] OR "Pain, Postoperative / surgery"[Mesh] OR Arthroplasty OR arthroplasty, replacement, knee OR Knee OR Knee arthroplasty OR osteoarthritis OR Pain OR Pain, postoperative OR replacement–total knee OR TKA OR Total knee arthroplasty

AND

"Analgesia"[Mesh] OR "Analgesics"[Mesh] OR "Analgesics / administration & dosage"[Mesh] OR "Analgesics / therapeutic use"[Mesh] OR "Anesthesia, Conduction"[Mesh] OR "Chronic Pain / drug therapy"[Mesh] OR "Pain / prevention & control"[Mesh] OR "Pain Management"[Mesh] OR "Pain Management / methods"[Mesh] OR "Pain, Postoperative / drug therapy"[Mesh] OR "Perioperative Care / methods"[Mesh] OR analgesia OR pain control OR pain management OR Postoperative analgesia

AND

"Analgesia / methods"[Mesh] OR "Analgesics, Non-Narcotic / therapeutic use"[Mesh] OR "Analgesics, Opioid"[Mesh] OR "Anesthesia, Epidural"[Mesh] OR "Anesthesia, Local"[Mesh] OR "Anesthesia, Spinal"[Mesh] OR "Anesthetics, Intravenous / therapeutic use"[Mesh] OR "Anesthetics, Local"[Mesh] OR "Autonomic Nerve Block / methods"[Mesh] OR "Catheterization, Peripheral / methods"[Mesh] OR "Drug Combinations"[Mesh] OR "Drug Therapy, Combination"[Mesh] OR "Femoral Nerve"[Mesh] OR "Infusion Pumps"[Mesh] OR "Infusions, Intravenous"[Mesh] OR "Injections"[Mesh] OR "Injections, Intra-Articular"[Mesh] OR "Lumbosacral Plexus"[Mesh] OR "Nerve Block"[Mesh] OR "Nerve Block / methods"[Mesh] OR "Sciatic Nerve"[Mesh] OR analgesia / methods OR Continuous OR Femoral block OR femoral nerve block OR Intra-articular OR iPACK OR Lateral femoral cutaneous nerve block OR LIA OR Local infiltration OR Local infiltration analgesia OR multimodal analgesia OR Multimodal infiltration OR Nerve block OR Periarticular drug injection OR periarticular infiltration OR periarticular injection OR Perineural catheters OR peripheral nerve block OR Single injection femoral nerve block OR Single shot

AND

"Pain Measurement"[Mesh] OR "Pain Measurement / methods"[Mesh] OR "Treatment Outcome"[Mesh] OR "Visual Analog Scale"[Mesh] OR outcomes OR VAS OR visual analogue score

SUMMARY

("Arthralgia / diagnosis"[Mesh] OR "Arthralgia / etiology"[Mesh] OR "Arthroplasty, Replacement, Hip"[Mesh] OR "Arthroplasty, Replacement, Knee"[Mesh] OR "Intraoperative Care"[Mesh] OR "Intraoperative Period"[Mesh] OR "Joint Capsule / innervation"[Mesh] OR

"Knee Joint"[Mesh] OR "Knee Joint / physiopathology"[Mesh] OR "Knee Joint / surgery"[Mesh] OR "Osteoarthritis, Knee / pathology"[Mesh] OR "Osteoarthritis, Knee / surgery"[Mesh] OR "Pain, Postoperative / surgery"[Mesh] OR Arthroplasty OR arthroplasty, replacement, knee OR Knee OR Knee arthroplasty OR osteoarthritis OR Pain OR Pain, postoperative OR replacement–total knee OR TKA OR Total knee arthroplasty) AND ("Analgesia"[Mesh] OR "Analgesics"[Mesh] OR "Analgesics / administration & dosage"[Mesh] OR "Analgesics / therapeutic use"[Mesh] OR "Anesthesia, Conduction"[Mesh] OR "Chronic Pain / drug therapy"[Mesh] OR "Pain / prevention & control"[Mesh] OR "Pain Management"[Mesh] OR "Pain Management / methods"[Mesh] OR "Pain, Postoperative / drug therapy"[Mesh] OR "Perioperative Care / methods"[Mesh] OR analgesia OR pain control OR pain management OR Postoperative analgesia) AND ("Analgesia / methods"[Mesh] OR "Analgesics, Non-Narcotic / therapeutic use"[Mesh] OR "Analgesics, Opioid"[Mesh] OR "Anesthesia, Epidural"[Mesh] OR "Anesthesia, Local"[Mesh] OR "Anesthesia, Spinal"[Mesh] OR "Anesthetics, Intravenous / therapeutic use"[Mesh] OR "Anesthetics, Local"[Mesh] OR "Autonomic Nerve Block / methods"[Mesh] OR "Catheterization, Peripheral / methods"[Mesh] OR "Drug Combinations"[Mesh] OR "Drug Therapy, Combination"[Mesh] OR "Femoral Nerve"[Mesh] OR "Infusion Pumps"[Mesh] OR "Infusions, Intravenous"[Mesh] OR "Injections"[Mesh] OR "Injections, Intra-Articular"[Mesh] OR "Lumbosacral Plexus"[Mesh] OR "Nerve Block"[Mesh] OR "Nerve Block / methods"[Mesh] OR "Sciatic Nerve"[Mesh] OR analgesia / methods OR Continuous OR Femoral block OR femoral nerve block OR Intra-articular OR iPACK OR Lateral femoral cutaneous nerve block OR LIA OR Local infiltration OR Local infiltration analgesia OR multimodal analgesia OR Multimodal infiltration OR Nerve block OR Periarticular drug injection OR periarticular infiltration OR periarticular injection OR Perineural catheters OR peripheral nerve block OR Single injection femoral nerve block OR Single shot) AND ("Pain Measurement"[Mesh] OR "Pain Measurement / methods"[Mesh] OR "Treatment Outcome"[Mesh] OR "Visual Analog Scale"[Mesh] OR outcomes OR VAS OR visual analogue score)
